# *Saccharomyces cerevisiae* boulardii Reduces the Deoxynivalenol-Induced Alteration of the Intestinal Transcriptome

**DOI:** 10.3390/toxins10050199

**Published:** 2018-05-15

**Authors:** Imourana Alassane-Kpembi, Philippe Pinton, Jean-François Hupé, Manon Neves, Yannick Lippi, Sylvie Combes, Mathieu Castex, Isabelle P. Oswald

**Affiliations:** 1 Toxalim (Research Centre in Food Toxicology), Université de Toulouse, INRA, ENVT, INP-PURPAN, UPS, BP.93173 F-31027 Toulouse CEDEX 3, France; philippe.pinton@inra.fr (P.P.); manon.neves@inra.fr (M.N.); yannick.lippi@inra.fr (Y.L.); 2 Hôpital d’Instruction des Armées—Centre Hospitalier Universitaire Cotonou Camp Guézo, Cotonou 01BP517, Benin; 3 Lallemand SAS, 19 rue des Briquetiers, BP 59, 31702 Blagnac CEDEX, France; jfhupe@lallemand.com (J.-F.H.); mcastex@lallemand.com (M.C.); 4 GenPhySE, Université de Toulouse, INRA, ENVT, 31320 Castanet Tolosan, France; sylvie.combes@inra.fr

**Keywords:** deoxynivalenol, *Saccharomyces cerevisiae* boulardii CNCM I-1079, intestine, transcriptome, inflammation, oxidative stress, lipid metabolism

## Abstract

Type B trichothecene mycotoxin deoxynivalenol (DON) is one of the most frequently occurring food contaminants. By inducing trans-activation of a number of pro-inflammatory cytokines and increasing the stability of their mRNA, trichothecene can impair intestinal health. Several yeast products, especially *Saccharomyces cerevisiae*, have the potential for improving the enteric health of piglets, but little is known about the mechanisms by which the administration of yeast counteracts the DON-induced intestinal alterations. Using a pig jejunum explant model, a whole-transcriptome analysis was performed to decipher the early response of the small intestine to the deleterious effects of DON after administration of *S. cerevisiae* boulardii strain CNCM I-1079. Compared to the control condition, no differentially expressed gene (DE) was observed after treatment by yeast only. By contrast, 3619 probes—corresponding to 2771 genes—were differentially expressed following exposure to DON, and 32 signaling pathways were identified from the IPA software functional analysis of the set of DE genes. When the intestinal explants were treated with *S. cerevisiae* boulardii prior to DON exposure, the number of DE genes decreased by half (1718 probes corresponding to 1384 genes). Prototypical inflammation signaling pathways triggered by DON, including NF-κB and p38 MAPK, were reversed, although the yeast demonstrated limited efficacy toward some other pathways. *S. cerevisiae* boulardii also restored the lipid metabolism signaling pathway, and reversed the down-regulation of the antioxidant action of vitamin C signaling pathway. The latter effect could reduce the burden of DON-induced oxidative stress. Altogether, the results show that *S. cerevisiae* boulardii reduces the DON-induced alteration of intestinal transcriptome, and point to new mechanisms for the healing of tissue injury by yeast.

## 1. Introduction

The intestine has been identified as one of the critical targets of the food-borne mycotoxins [1,2]. Trichothecene (TCT) mycotoxin deoxynivalenol (DON) causes intestinal immune alterations which mimick inflammatory bowel diseases [3,4,5]. At the molecular level, the binding of DON or other TCTs to the ribosome activates mitogen-activated kinases (MAPKs), and also induces trans-activation of a number of pro-inflammatory cytokines, and an increase of the stability of their mRNA [6,7]. We showed that the frequently reported co-exposure to DON and other TCT could result in a synergistic inflammatory response of the intestinal tissue [8,9,10].

Among the ways to tackle the variety of factors threatening intestinal health, probiotics are considered good candidates, since they not only suppress the growth and binding of pathogenic bacteria, as well as improving the barrier function of the intestinal epithelium, but also tune the immune activity of the host [11,12]. Several yeast products are listed under the EC Regulation N.1831/2003 as feed additives for pigs in the EU; *Saccharomyces cerevisiae* in particular has demonstrated its potential for improving enteric health of piglets [13,14]. It was also recently shown that the yeast culture additive reduces immune and liver damage in pigs induced by a DON and aflatoxin contaminated diet [15].

Little is known about the mechanisms by which *S. cerevisiae* improves intestinal health. Preliminary in vitro data obtained in human monocytic, colonic, and gastric epithelial cells suggest that *S. cerevisiae* boulardii decreases inflammation by inhibiting NF-κB-mediated *IL-8* gene expression [16]. Regarding DON-induced alterations, *S. cerevisiae* boulardii decreases the activation of p38 MAPK pathway, and reduces the expression of downstream inflammatory cytokines; this yeast also promotes the expression of anti-apoptotic genes, and inhibits apoptosis in porcine alveolar macrophage cells [17].

In order to understand the early response of the small intestine to the administration of *S. cerevisiae* boulardii in response to the deleterious effects of DON, a whole transcriptome analysis of intestinal tissue was performed. Pre-treatment of the intestinal tissue with the yeast significantly reduced the global impact of DON on the transcriptome, and reversed prototypical inflammation signaling pathways including NF-κB and p38 MAPK. The yeast also restored the lipid metabolism signaling pathway and reverted the down-regulation of the antioxidant action of the vitamin C signaling pathway.

## 2. Results and Discussion

### 2.1. S. cerevisiae boulardii Reduces the Transcriptomic Impact of DON on the Intestine

To analyze the ability of *S. cerevisiae* boulardii to counteract the intestinal effect of DON, a pan genomic analysis of the transcriptomic fingerprint was conducted in control jejunal explants, explants treated with the yeast, and explants challenged with 10 μM DON in the presence, or absence, of *S. cerevisiae* boulardii. In human gut, concentrations of 0.16–2 μg DON/mL (0.5–7 μM) are considered realistic [8]. The lowest concentration value corresponds to the mean estimated daily intake of French adult consumers on a chronic basis. The highest concentration value simulates levels that can be reached after the consumption of heavily contaminated food, as can be occasionally encountered. Animals, especially pigs, can be exposed to higher concentrations of DON. Assuming that DON is ingested in one meal, diluted in 1 L of gastrointestinal fluid, and is totally bio accessible, the in vitro concentration of 10 μM used in this study corresponds to feed contaminated with 3 mg DON/Kg [18]. In a recent scientific opinion, EFSA reported levels of DON in feed grains of up to 9.5 mg/kg [19]. Regarding the use of *S. cerevisiae* boulardii, the EFSA expert panel concluded that the additive has the potential to be efficacious in weaned piglets at a dose of 2 × 10^9^ CFU/kg feed [20]. The yeast concentration used in this study was chosen to reflect the recommended dose.

**Figure 1:**
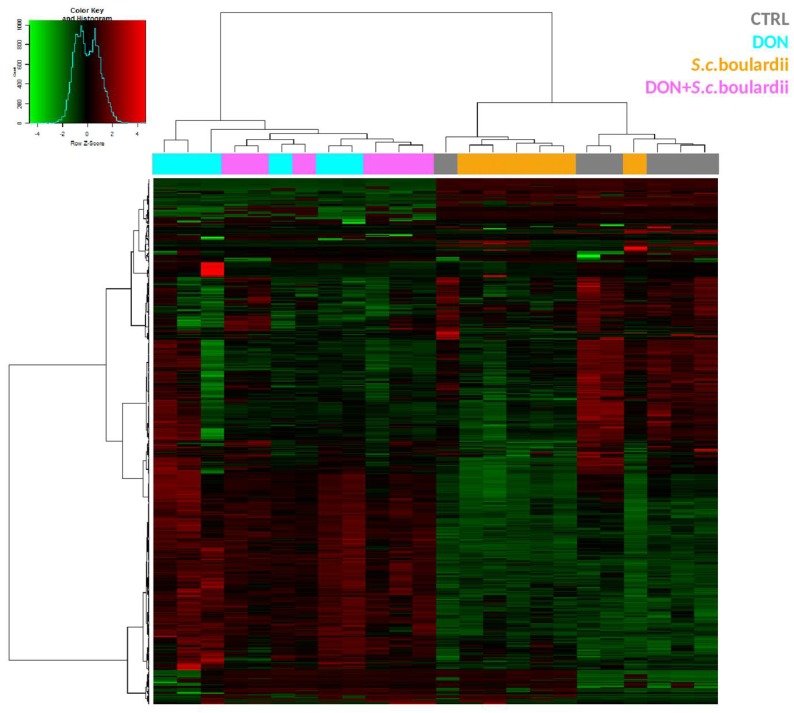
Gene expression profiles of intestinal explants exposed to DON or FX. Heatmap representing differentially expressed probes between the control, DON, *S. cerevisiae* boulardii and DON + *S. cerevisiae* boulardii conditions. Jejunal explants from six different animals were exposed for four hours to DMSO (vehicle), or treated with a *S. cerevisiae* boulardii suspension, or exposed to 10 μM DON. Gene expression was analyzed with a 60 K microarray. Red and green indicate values above and below the mean (average Z-score), respectively. Black indicates values close to the mean.

**Figure 2:**
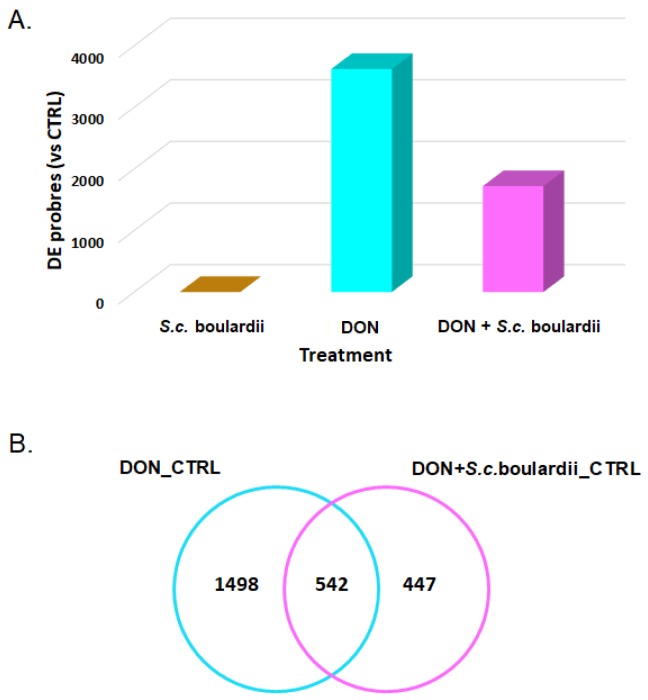
(**A**) Differentially expressed probes on the microarray for the yeast, mycotoxin, and yeast administration followed by mycotoxin exposure conditions; (**B**) Venn diagram of differentially expressed genes in the intestinal tissue after DON exposure or *S. cerevisiae* boulardii administration followed by DON exposure. CTRL represents DMSO control.

A total of 4398 transcripts, out of the 40,726 tested, were differentially expressed in at least one of the treatments when compared to control samples (adjusted *p* value < 0.05). As expected, and as shown on the heatmap (Figure 1), exposure of the intestinal tissue to DON resulted in clear alteration of the transcriptome. Hierarchical clustering indicated that control and *S. cerevisiae* boulardii-treated intestinal tissue samples separated from DON and DON + yeast treated groups. In comparison with the control, 3619 probes—corresponding to 2771 genes—were differentially expressed (DEG) (adjusted *p* value < 0.05) following exposure to DON, with fold-change values ranging from −3.43 to 11.44. No DEG was observed for the yeast condition (Figure 2A).

The top-regulated genes are listed in Table 1. As previously reported, the top-up regulated genes in intestinal explants exposed to DON were mainly related to inflammation [10,21,22]. The most up-regulated genes include interleukin 1 alpha and beta (*IL-1α* and *IL-1β*) and interleukin 22 (*IL-22*), the macrophage inflammatory proteins genes *MIP-2* alpha and *MIP-3* alpha (also known as *CXCL2* and *CCL20*), the receptor for MIP-3 beta chemokine gene (*CCR7*), the positive regulatory domain I-binding factor 1 gene (*PRDM1*) which drives the maturation of B-lymphocytes into Ig secreting cells, and the A disintegrin and metalloproteinase with thrombospondin motifs gene (*ADAMTS*) which may be associated with various inflammatory process. The amphiregulin gene (*AREG*), which can be expressed by multiple populations of activated immune cells under inflammatory conditions, was also up-regulated. Other genes in the top up-regulated category included *FOSL-1*, which regulates cell proliferation, differentiation, or transformation, *PLK-2*, which may play an important role in cells undergoing rapid cell division, and the transcriptional co-activator/repressor *IFRD1*, which controls the growth and differentiation of specific cell types.

The treatment of the intestinal explants with *S. cerevisiae* boulardii prior to DON exposure decreased the number of DEGs by 50%, as only 1384 genes (1718 probes) were differentially expressed in DON + yeast treated explants (Figure 2A). Nevertheless, the top up-regulated genes in the jejunal tissue treated with either DON alone, or *S. cerevisiae* boulardii prior to DON exposure, were very similar, and inflammation remained the hallmark of the intestinal transcriptome. It is worthy of note that, except for growth arrest and DNA damage-inducible protein gamma gene (*GADD45G*), the 15 other genes expressed in both experimental conditions were slightly less expressed in the DON + yeast condition (Table 1).

Microarray data validation was performed by quantitative real-time polymerase chain reaction (qPCR) on a set of 12 DE genes in DON and DON + yeast conditions. A significant Spearman correlation coefficient (r = 0.896) was observed between the two techniques (Supplementary Figure S1), confirming the reliability of the transcriptome analyses.

### 2.2. Functional Analysis of the Intestinal Transcriptome Modulation by DON and/or S. cerevisiae boulardii

To further investigate the protective effect of *S. cerevisiae* boulardii, a comparative functional analysis of DEGs in DON and DON + yeast conditions was performed using the core analysis comparative function of Ingenuity Pathway Analysis (IPA) software.

**Figure 3:**
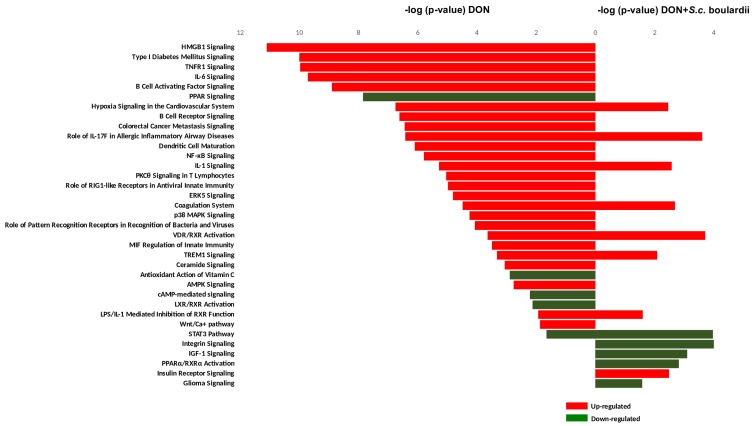
Top canonical pathways significantly modulated in DON and DON + *S. cerevisiae* boulardii conditions. Statistical significance of pathway modulation was calculated via a right-tailed Fisher’s exact test in IPA. Only pathways that presented a −log *p*-values exceeding 1.30 and −2 > IPA Z-score > +2 were preserved.

Figure 3 shows that 32 canonical pathways were significantly affected in the jejunal tissue exposed to DON (*p*-value < 0.05, −2 > IPA Z-score > +2). As previously described, the foremost up-regulated signaling pathways in the DON condition, including HMGB1 signaling, IL-6, IL1, TNFR1, p38 MAPK, and TREM1 signaling, were related to immunity/inflammation [21]. The B-cell activating factor, B-cell receptor signaling, and the NF-κB signaling pathway, which not only regulates inflammation and immunity, but also cell survival and cell proliferation, were also up-regulated by DON. Likewise, intestinal exposure to DON resulted in the down regulation of the PPAR and the LXR/RXR activation signaling pathways, which control diverse aspects of cholesterol and fatty acid homeostasis [21,23]. Lastly, a DON-induced oxidative stress in the intestinal tissue was reflected by down-regulation of the antioxidant action of the vitamin C signaling pathway.

Applying *S. cerevisiae* boulardii to the jejunal tissue prior to DON exposure reduced the number of activated canonical pathways from 32 to 13 (Figure 3). In fact, only eight out of the 32 canonical pathways triggered by DON really withstood the protective effects of *S. cerevisiae* boulardii, and five new signaling pathways emerged from a set of DEGs specific to the interaction between DON and yeast on the treated explants (Figure 2B and Figure 3). The pathways expressed in both DON and yeast + DON conditions were the hypoxia signaling, IL-1 signaling, TREM1 signaling, STAT3, the VDR/RXR activation, the LPS/IL-1 mediated inhibition of RXR function, the coagulation system pathway, and the IL-17F pathway in allergic inflammatory diseases. The interaction between DON and *S. cerevisiae* boulardii resulted in the down-regulation of integrin signaling, IGF-1 signaling, glioma signaling, and the PPARa/RXRa activation pathway, as well as the up-regulation of insulin receptor signaling; in contrast, none of these pathways was affected when individually exposed to DON or *S. cerevisiae* boulardii.

Aside from these few cases, the shutdown of the 24 other DON-triggered signaling pathways is evidence that the yeast administration significantly restrained the detrimental effects of this mycotoxin on the intestine. The application of *S. cerevisiae* boulardii was particularly effective on signaling pathways associated with (i) inflammation, (ii) oxidative stress, and (iii) lipid metabolism.

### 2.3. S. cerevisiae boulardii Reduces the Pro-Inflammatory Effects of DON

Only four out of 19 inflammation/immunity signaling pathways triggered by DON were still observed in explants treated with *S. cerevisiae* boulardii prior to DON exposure. These four signaling pathways were IL-1 and TREM signaling, the LPS/IL-1 mediated inhibition of RXR function, as well as the role of IL-17 in allergic inflammatory disease pathway. Treatment with *S. cerevisiae* boulardii prevented the triggering of other inflammation pathways including HMGB1 signaling, TNFR1 signaling, and the IL-6 signaling pathway, as well as the pivotal NF-kB signaling and p38 MAPK signaling pathways.

HMGB1 belongs to a family of endogenous compounds termed “alarmins”, which can be released in the extracellular milieu during states of cellular stress or injury leading to infectious or non-infectious conditions [24]. IL-6 is generated upon activation of the pattern recognition receptors (PRRs) by pathogen-associated molecular patterns (PAMPs) or damage-associated molecular patterns (DAMPs), in the case of infectious lesion or tissue damage [25]. The DON-induced ribotoxic stress has been previously linked with ribosomal RNA cleavage, which could be the starting point of a DAMP-mediated PRR activation of the IL-6 and HMGB1 signaling pathways in the intestine [26]. The hypothesis of cytosolic sensing of cleaved RNA is also supported by the triggering of both a signaling pathway linked with the role of pattern recognition receptors in the recognition of bacteria and viruses, and even more, by another pathway associated with the role of RIG1-like receptors in antiviral innate immunity in presence of DON (Figure 3).

**Figure 4:**
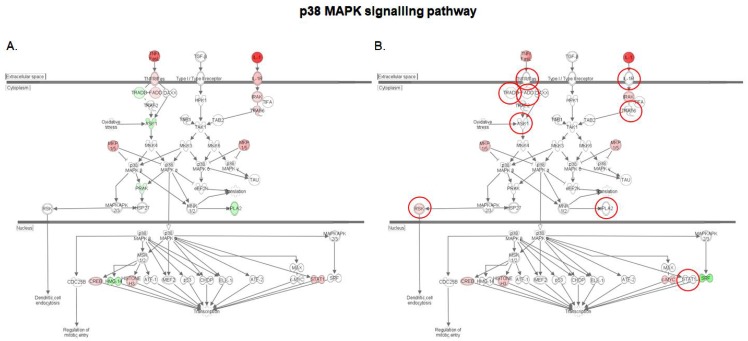
Expression patterns of genes of the p38MAPK signaling pathway in the intestinal tissue. (**A**) Intestinal tissue exposed to 10 μM DON; (**B**) Intestinal tissue exposed to *S. cerevisiae* boulardii and 10 μM DON. Red nodes represent up-regulated genes, and green nodes represent down-regulated genes identified in our differential expression analysis. Darker node colors indicate more extreme (high or low) up- or down-regulation of the respective gene. White nodes represent genes involved in the signaling pathway that were not identified in the microarray analysis. The red circles indicate genes showing different expression pattern in both conditions.

The p38 MAPK pathway is a key regulator of pro-inflammatory cytokines biosynthesis at the transcriptional and translational levels, which makes it critical for normal immune and inflammatory response [27]. In isolated porcine alveolar macrophage cells, *S. cerevisiae* boulardii was shown to attenuate p38 MAPK signaling by alleviating DON-induced phosphorylation and mRNA expression of p38 MAPK protein [17]. A comparison of the activation patterns of the different genes involved in the p38 MAPK in both DON-exposed tissue and *S. cerevisiae* boulardii treated tissue before DON exposure (Figure 4) shows that the remediation action of *S. cerevisiae* boulardii involves many more mechanisms in all the cellular compartments. First, the yeast counteracts the DON-induced upregulation of the transmembrane receptors IL-1R and TNFR/Fas, as well as its adaptor molecule FADD, both of which transduce the triggering signals delivered by IL-1 cytokine and Fas ligand. In the cytosolic compartment, pre-treatment with *S. cerevisiae* boulardii before DON exposure also leads to restoration of the DON-induced downregulation of apoptosis signal-regulating kinase 1 (ASK1) and a member of the cytosolic phospholipase A2 family (cPLA2), and up-regulation of the TNF receptor associated factor 6 (TRAF6). Recent studies revealed the involvement of ASK1 in ROS- or ER stress related diseases, suggesting that ASK1 could be a therapeutic target [28]. The members of the cPLA2 family catalyze the hydrolysis of phospholipids to yield fatty acids and lysophospholipids, and are involved in inflammation by driving arachidonic acid metabolism [29]. Finally, in the intestinal tissue, *S. cerevisiae* boulardii was also seen to downscale the DON-induced activation of the signal transducer and activator of transcription 1 (STAT1), which translocates to the nucleus to promote expression of cytokine response genes [30].

**Figure 5:**
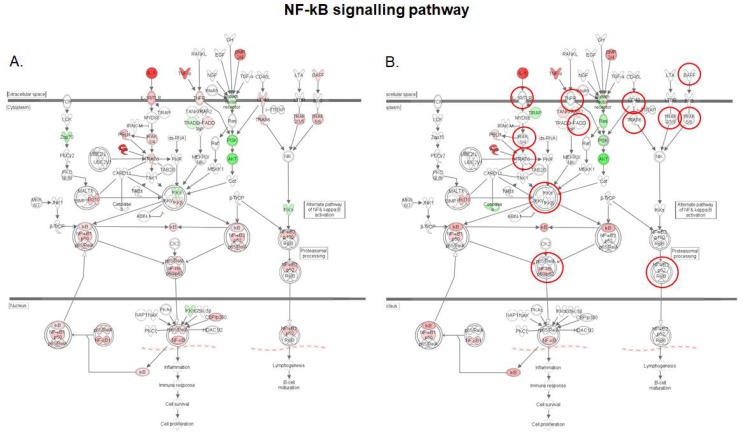
Expression patterns of genes of the NF-κB signaling pathway in the intestinal tissue. (**A**) Intestinal tissue exposed to 10 μM DON; (**B**) Intestinal tissue exposed to *S. cerevisiae* boulardii and 10 μM DON. Red nodes represent up-regulated genes, and green nodes represent down-regulated genes identified in our differential expression analysis. Darker node colors indicate more extreme (high or low) up- or down-regulation of the respective gene. White nodes represent genes involved in the signaling pathway that were not identified in the microarray analysis. The red circles indicate genes showing different expression pattern in both conditions.

Figure 5 summarizes the effect of DON in the presence, or absence, of *S. cerevisiae* boulardii on the NF-κB signaling pathway. Activation of NF-κB signaling via canonical or alternative pathways regulates various biological processes, including immune response, inflammation, cell growth and survival, and development [31]. We observed that upon intestinal exposure to DON, both canonical and alternative NF-κB signaling pathways were activated. IL-1, TNF-α and bone morphogenetic protein 2 (BMP2) primary signals converged to the nuclear translocation of the heterodimeric NF-κB complex p50/RelA via activation of the catalytic IκB kinase subunits IKKα and IKKβ, while the B-cell activating factor (BAFF) signal led to upregulation and subsequent nuclear translocation of p52/RelB dimer. By contrast, neither p50/RelA, nor p52/RelB complexes showed up-regulation of gene expression when the intestinal explants were treated with *S. cerevisiae* boulardii prior to DON exposure (supplementary Table S1). Interestingly, administration of *S. cerevisiae* boulardii counteracts upstream, the DON-induced upregulation of transmembrane receptors IL-1R/TLR, TNFR and CD40 and adaptor molecules FADD, TRADD and TRAF, on the one hand, and on the other hand, the DON-induced upregulation of BAFF. This suggests that *S. cerevisiae* boulardii may prevent the activation of the canonical pathway of NF-κB signaling by altering the transduction of IL-1 and TNF-α primary signals, and the activation of the alternative pathway by suppressing the initial BAFF signal.

To summarize, the administration of *S. cerevisiae* boulardii prevented the intestinal tissue from the onset of several signaling pathways driving inflammation that is the hallmark of the low-dose exposure to DON and other trichothecene mycotoxins.

### 2.4. S. cerevisiae boulardii Reverses the Effect of DON on the Antioxidant Action of Vitamin C

**Figure 6:**
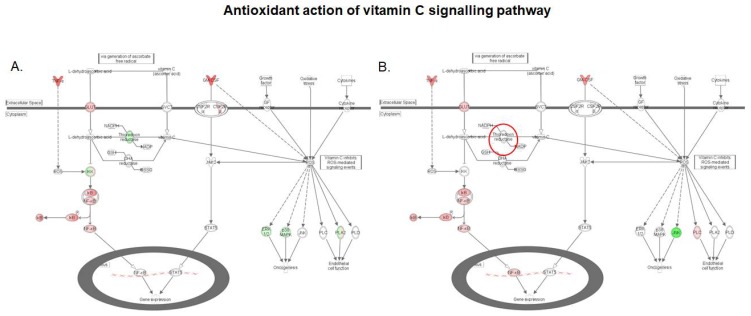
Expression patterns of genes of the antioxidant action of vitamin C signaling pathway in the intestinal tissue. (**A**) Intestinal tissue exposed to 10 μM DON; (**B**) Intestinal tissue exposed to *S. cerevisiae* boulardii and 10 μM DON. Red nodes represent up-regulated genes, and green nodes represent down-regulated genes identified in our differential expression analysis. Darker node colors indicate more extreme (high or low) up- or down-regulation of the respective gene. White nodes represent genes involved in the signaling pathway that were not identified in the microarray analysis. The red circles indicate genes showing different expression pattern in both conditions.

The antioxidant action of the vitamin C signaling pathway was down-regulated in the intestinal tissue following exposure to DON (Figure 6). Feeding a DON–contaminated feed to broiler chickens has previously been shown to significantly up-regulate the expression of two sensitive markers of oxidative injury, hypoxia inducible factor 1 subunit alpha (HIF-1α), and heme-oxygenase (HMOX), in intestinal tissue [32]. Oxidative stress is one of the most important underlying mechanisms of the toxicity of DON in eukaryotic cells, and two mechanisms (generation of reactive oxygen species (ROS) and alteration in antioxidant status) have been reported for this mycotoxin [33,34]. Both mechanisms are represented in Figure 6. The DON-induced cytokine signal transduction in the intestinal tissue could generate ROS, while the antioxidant action of vitamin C is repressed. The protective effect of vitamin C as a free radical scavenger warrants its use in the prevention of DON-mediated oxidative stress [35]. Vitamin C can enter the cell directly via sodium-dependent ascorbate co-transporters (SVCT); alternatively, GLUT transporters can carry dehydroascorbic acid that will be reduced to vitamin C by enzymes such as thioredoxin reductase (TXN) and dehydroascorbic acid reductase (DHA), once inside the cell [36]. Hence, by downregulating the TXN gene (Figure 6), DON could impair the conversion of dehydroascorbic acid, and limit intracellular levels of vitamin C, leading to lowered capacity for the inhibition of ROS-mediated events.

As shown by the microarray data in Table 1, and confirmed by the qRT-PCR data in Table 2 and supplementary Table S2, the pre-treatment of the intestinal tissue with *S. cerevisiae* boulardii had a limited effect on the DON induced up-regulation of cytokine expression, which means that the ROS generation was preserved. Nonetheless, comparison of gene activation patterns in the antioxidant action of vitamin C signaling pathway in the DON and DON + *S. cerevisiae* boulardii conditions indicates that the yeast treatment counteracted the DON-induced down-regulation of TXN (Figure 6). This highlights the fact that, although *S. cerevisiae* boulardii could not protect the intestinal tissue from ROS generation, the probiotic helps restore intracellular vitamin C, and could downscale the burden of the DON-induced oxidative stress via ROS generation.

### 2.5. S. cerevisiae boulardii Restores the Lipid Metabolism Altered by DON

**Figure 7:**
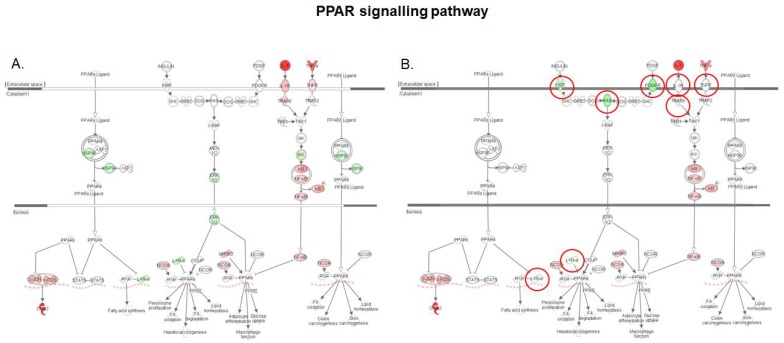
Expression patterns of genes of the PPAR signaling pathway in the intestinal tissue. (**A**) Intestinal tissue exposed to 10 μM DON; (**B**) Intestinal tissue exposed to *S. cerevisiae* boulardii and 10 μM DON. Red nodes represent up-regulated genes, and green nodes represent down-regulated genes identified in our differential expression analysis. Darker node colors indicate more extreme (high or low) up- or down-regulation of the respective gene. White nodes represent genes involved in the signaling pathway that were not identified in the microarray analysis. The red circles indicate genes showing different expression pattern in both conditions.

The functional analysis of the intestinal transcriptome upon exposure to DON revealed repression of both PPAR signaling and the LXR/RXR activation which control lipid metabolism (Figure 7). The PPAR/RXR and LXR/RXR heterodimers act as sensors of intracellular lipid metabolism, and thereby, regulate diverse aspects of cholesterol and fatty acid homeostasis [23]. PPARγ is a highly expressed nuclear receptor in macrophages, and its activation increases *LXRα* expression, which in turn transactivates target genes and reduces cell cholesterol levels. As a consequence, deficiency in PPAR signaling is pivotal for the establishment of the foam cells of atherosclerotic lesions [37]. As shown in Figure 7, on the one hand, intestinal exposure to DON repressed *PPARγ* via NF-κB signaling, and on the other hand, down-regulated the expression of *LXRα* gene. By contrast, the DON-induced down regulation of *LXRα* was prevented by the pre-treatment of intestinal tissue with *S. cerevisiae* boulardii along with the canceling of signal transduction at an early-stage of the NF-κB pathway.

Interestingly, expression of the insulin receptor gene and of the platelet-derived growth factor receptor gene (*PDGF*) was also down-regulated. As a phosphoprotein, the transcriptional activity of PPARγ is affected by the extracellular receptor kinase-mitogen activated protein kinase (ERK-MAPK), and thereby its constitutive activators insulin and PDGF [38]. Phosphorylation by ERK has been shown to decrease the transcriptional activity of PPARγ [39,40]. Hence, the down-regulation of the insulin and PDGF receptors observed in tissue treated with yeast prior to exposure to DON could limit the basal inactivation of PPARγ, and function as a potentiating factor for recovery from DON-suppressed PPAR functions.

## 3. Conclusions

The aim of the present study was to investigate the effects of *S. cerevisiae* boulardii as a strategy to alleviate the effect of DON. To this end, a transcriptional analysis of intestinal explant was performed. On its own, *S. cerevisiae* boulardii induced no differential gene expression in the intestine. However, application of the yeast significantly reduced the global impact of DON on the transcriptome. Prototypical signaling pathways linked to inflammation and immunity, including the NF-κB and the p38 MAPK, triggered by DON were reversed by the *S. cerevisiae* boulardii treatment, although the yeast showed limited efficacy toward some other signaling pathways. Transcriptomic data also suggest that *S. cerevisiae* boulardii reduces the burden of the global DON-induced oxidative stress and restores the PPAR signaling functions which control lipid homeostasis. How these striking observations, made in an oversimplified explant model, apply at a functional level in vivo still remains to be investigated.

## 4. Materials and Methods

### 4.1. Toxin

Purified deoxynivalenol (DON) purchased from Sigma (Saint Quentin Fallavier, France) was dissolved to 10 mM in dimethylsulfoxide (DMSO), and stored at −20 °C before dilution in complete culture media.

### 4.2. Yeast Strain and Culture

Industrial dry yeast Levucell SB20 (*Saccharomyces cerevisiae* boulardii CNCM I-1079) from Lallemand Animal Nutrition (Blagnac, France) was rehydrated (10 g/100 g) in a suspension medium (NaCl, K_2_HPO_4_, KH_2_PO_4_, casein peptone and Tween 80) and allowed to rehydrate for 20 min with gentle agitation (120 rpm) in a shaking water bath at 37 °C. Serial dilutions were performed in peptone saline water (NaCl, casein peptone), and a 1/1000 dilution containing 4 × 10^6^ CFU/mL of live cells was used. The yeast solutions were prepared just before use. Whole culture material was used in this experiment, since it is established that the effects of *S. cerevisiae* boulardii on the host result from cell to cell interactions, as well as from interactions between yeast secreted molecules and epithelial cells [41,42].

### 4.3. Culture of Jejunum Explants

Jejunal explants were obtained from 5-week old crossbred castrated male piglets, as described previously [7,43,44]. Briefly, 5 cm middle jejunum segments were collected in complete William’s Medium E (Sigma, Saint Quentin Fallavier, France). Four to six washes were performed with William’s Medium E. After removing external *tunica muscularis*, each jejunum segment was opened longitudinally, and pieces of 6 mm diameter were obtained with biopsy punches (Kruuse, Centravet, Dinan, France). Two explants/wells were deposited villi upward on 1-cm^2^ biopsy sponges (Medtronic, Minneapolis, MN, USA) in six well plates (Cellstar, Greiner Bio-One, Frickenhausen, Germany) containing the control medium, supplemented or not with *S. cerevisiae* boulardii containing medium. The culture medium contained 100 U/mL penicillin, 100 μg/mL streptomycin, and 50 μg/mL gentamycin (Eurobio, Courtaboeuf, France). All these operations were achieved in less than one hour after the piglets were euthanized.

The experiment was conducted according to the guidelines of the French Ministry of Agriculture for animal research. All animal experimentation procedures were approved by the Ethics committee of Pharmacology-Toxicology of Toulouse-Midi-Pyrénées in animal experimentation (Toxcométhique), N° TOXCOM/0017/IO PP, in accordance with the European Directive on the protection of animals used for scientific purposes (Directive 2010/63/EU). The date of approval is 1 September 2017. The three authors (I.A.K, I.P.O. and P.P) have an official agreement with the French Veterinary Services allowing animal experimentation. The explants were pre-treated, or not, with 75 μL of the *S. cerevisiae* boulardii. I-1079 (which corresponds to 3 × 10^5^ colony forming units) for 30 min, and then challenged with 10 μM DON for 4 h at 39 °C. The dose of DON and *S. cerevisiae* boulardii I-1079 were chosen to reflect doses in feed equivalent to 3 ppm of DON and 2 × 10^9^ CFU/kg of feed of *S. cerevisiae* boulardii I-1079, respectively. A toxin vehicle (DMSO) condition, and an *S. cerevisiae* boulardii I-1079 condition, were used as controls. After incubation, treated explants were stored at −80 °C before RNA extraction.

### 4.4. RNA Extraction

Jejunal explants were lysed in 1 mL of Extract All reagent (Eurobio, Les Ulis, France) with ceramic beads (Bertin Technologies, Saint Quentin en Yvelines, France). Total RNA was extracted according to the manufacturer’s recommendations, as previously described [45,46]. The RNA concentration was determined by measuring the optical density at 260 nm, and the RNA integrity was assessed using both NanoDrop spectrophotometric analysis (Labtech International, Paris, France) and Agilent capillary electrophoresis (Agilent 2100 Bioanalyzer, Agilent Technologies Inc., Santa Clara, CA, USA). The mean (±SD) RNA integrity number (RIN) of these mRNA preparations was 6.85 (±0.8).

### 4.5. Microarray Processing and Functional Analysis of Expressed Genes

The microarray GPL16524 (Agilent technology, Santa Clara, CA, USA, 8 × 60 K) used in this experiment consisted of 43,603 spots derived from the 44 K (V2:026440 design) Agilent porcine-specific microarray [21]. This microarray was enhanced with 9532 genes from adipose tissue, 3776 genes from the immune system, and 3768 genes from skeletal muscle. A total of 24 samples (6 replicates per treatment group) were processed. For each sample, cyanine-3 (Cy3)-labeled cRNA was prepared from 200 ng of total RNA using the One-Color Quick Amp Labeling kit (Agilent, Santa Clara, CA, USA), according to the manufacturer’s instructions, followed by the Agencourt RNAClean XP (Agencourt Bioscience Corporation, Beverly, MA, USA). Approximately 600 ng of Cy3-labeled cRNA was hybridized onto the SurePrint G3 Porcine GE microarray (8 × 60 K), following the manufacturer’s instructions. Immediately after washing, the slides were scanned on an Agilent G2505C Microarray Scanner using Agilent Scan Control A.8.5.1 software, and the fluorescence signals were extracted using the Agilent Feature Extraction software v10.10.1.1 with the default parameters. The microarray data were analyzed using R (www.r-project.org, R v. 3.1.2) and the Bioconductor packages (www.bioconductor.org, v 3.0), as described in GEO entry GSE97821, which also contains all the details of the experiments.

Network analysis and functional analysis of the DE genes were performed using the Ingenuity Pathway Analysis tool (IPA, http://www.ingenuity.com) to identify gene networks and signaling pathways affected by each treatment. The IPA output included statistical assessment of the significance of gene networks and signaling pathways based on Fisher’s exact test; only networks and pathways that presented a *p* value < 0.05 or a −log *p* value exceeding 1.30 (FDR *q*-values < 0.05) and a Z-score with an absolute value ≥ 2 were retained.

### 4.6. Quantitative Real-Time Polymerase Chain Reaction (qRT-PCR) Analysis

The reverse transcription and real-time qPCR steps were performed on total RNA samples (*n* = 6 per treatment group), as previously described [47]. The primers for selected differentially expressed genes on the microarray (Supplementary Table S3) were designed using Primer Express Software, and purchased from Invitrogen (Invitrogen, Life Technologies Corporation, Paisley, UK). Non-reverse transcribed RNA was used as the non-template control for verification of the genomic DNA amplification signal. The specificity of the qPCR products was assessed at the end of the reactions by analyzing the dissociation curves. The expression stability of five candidate reference genes, (Cyclophylin A (*CycloA*), β-actin, β2-microglobulin, Ribosomal Protein L32 (*RPL32*), and Hypoxanthine Phosphoribosyl transferase 1 (*HPRT-1*)) across different experimental samples has been investigated; it was demonstrated that *RPL32* showed the highest stability (SD = 0.82; r = 0.971) [21]. *RPL32* and *CycloA* were chosen as housekeeping genes, and the 2-∆∆Ct method was used to calculate the fold change in gene expression. For comparison with microarray data, the Spearman correlation between the microarray Log2 (FC) and qPCR Log2 (2-∆∆Ct) values was calculated.

### 4.7. Statistical Analysis

The microarray data were analyzed using the R Bioconductor packages and the limma lmFit function, as previously described [21]. Probes with adjusted *p* values ≤ 0.05 (FDR correction using the Benjamini Hochberg procedure) were differentially expressed between the treated and control conditions. Hierarchical clustering was applied to the samples and the probes, using the 1-Pearson correlation coefficient as the distance and Ward’s criterion for agglomeration, and was then illustrated as a heatmap presenting gene expression profiles of selected regulated genes.

For gene expression quantification by qRT-PCR, the mRNA expression of the target genes was normalized to the expressed housekeeping genes using REST© 2009 software (Qiagen, Valencia, CA, USA), which uses the pair-wise fixed reallocation randomization test as the statistical model [10].

## Figures and Tables

**Table 1:** Top-20 up- and down-regulated genes in the DON and the DON + *S. cerevisiae* boulardii conditions of the microarray experiment.

	DON			DON + *S. cerevisiae* boulardii		
	Gene Symbol	Fold Change *	Adjusted *p* Value	Gene Symbol	Fold Change *	Adjusted *p* Value
Up–regulated	*IL-1β*	11.37	6.27 × 10^−13^	*IL-1β*	9.56	6.01 × 10^−12^
	*IL-22*	5.62	3.24 × 10^−8^	*IL-22*	5.56	7.73 × 10^−8^
	*NOR-1*	5.09	1.07 × 10^−9^	*PTGS2*	3.96	1.32 × 10^−6^
	*PTGS2*	5.01	3.63 × 10^−8^	*IL-1α*	3.96	2.90 × 10^−8^
	*CXCL2*	4.65	0.00045897	*CCL20*	3.96	3.85 × 10^−7^
	*IL-1α*	4.17	5.78 × 10^−9^	*NOR-1*	3.88	7.73 × 10^−8^
	*CCL20*	4.17	8.96 × 10^−8^	*CXCL2*	3.22	0.01419023
	*CCR7*	3.36	1.36 × 10^−7^	*IL8*	2.95	0.0004445
	*HAMP*	3.34	2.46 × 10^−5^	*ARX 10G*	2.87	7.48 × 10^−7^
	*PLK2*	3.28	1.02 × 10^−11^	*PRDM1*	2.86	8.79 × 10^−12^
	*AREG*	3.26	7.49 × 10^−8^	*FOSL1*	2.82	0.00026329
	*PRDM1*	3.17	6.27 × 10^−13^	*GADD45G*	2.7	9.54 × 10^−6^
	*FOSL1*	3.11	4.71 × 10^−5^	*CCR7*	2.63	1.35 × 10^−5^
	*CSF2*	2.93	4.60 × 10^−5^	*PLK2*	2.6	2.32 × 10^−9^
	*ADAMTS1*	2.92	9.33 × 10^−10^	*GADD45A*	2.6	2.12 × 10^−6^
	*CRSP-2*	2.9	1.31 × 10^−5^	*PRDM1*	2.59	9.84 × 10^−10^
	*CD274*	2.74	3.47 × 10^−7^	*ATF3*	2.57	4.04 × 10^−6^
	*RRAD*	2.73	0.00150172	*PLK2*	2.5	3.89 × 10^−9^
	*IFRD1*	2.66	1.47 × 10^−8^	*RND1*	2.48	3.07 × 10^−6^
	*GADD45G*	2.64	7.57 × 10^−6^	*HAMP*	1.31	0.001954
Down–regulated	*CTBS*	−3.43	0.0323898	*ACTN2*	−4.83	0.03632277
	*C1QTNF3*	−2.38	0.02819327	*ACTA1*	−4.28	0.03241036
	*PDLIM3*	−2.2	0.04545171	*TPM2*	−2.88	0.04875964
	*BTC*	−2	0.04718046	*TPM1*	−2.81	0.04897366
	*TMEFF2*	−1.97	0.02364126	*MYL9*	−2.66	0.04636262
	*HAND1*	−1.89	0.04906228	*LIMS2*	−2.57	0.04897366
	*SMPX*	−1.82	0.01890697	*MYLK*	−2.53	0.04955867
	*BPI*	−1.76	0.03066332	*C1QTNF3*	−2.49	0.03403947
	*LYZ*	−1.72	0.02546107	*SPOCK3*	−2.41	0.02910998
	*LECT1*	−1.7	0.04314249	*BTC*	−2.26	0.04788811
	*ASPN*	−1.67	0.04519455	*TPM1*	−2.23	0.04179965
	*OGN*	−1.66	0.03041459	*HAND1*	−2.2	0.02447994
	*RYR2*	−1.64	0.03593429	*FLNA*	−2.1	0.03855459
	*DSTN*	−1.64	0.00936255	*TMX 10FF2*	−2.03	0.02907563
	*FHL1C*	−1.63	0.0438132	*CPXM2*	−2.02	0.02989774
	*CXCL12*	−1.63	0.01364805	*DNAJA4*	−1.94	0.01272008
	*CYP4F2*	−1.59	0.04673074	*PRUNX 102*	−1.86	0.0392422
	*ITGB1BP2*	−1.59	0.0354242	*SMARCD3*	−1.83	0.04490504
	*ACTB*	−1.59	0.02928137	*FHL1C*	−1.81	0.02399939
	*MATN2*	−1.59	0.0065206	*ATP2B4*	−1.81	0.04252773

* Fold change compared to DMSO control.

**Table 2:** Expression of key inflammatory genes in the DON and the DON + *S. cerevisiae* boulardii conditions in microarray and qRT-PCR experiments.

	DON				DON + *S. cerevisiae* boulardii			
Gene Symbol	FC Microarray *	Adjusted *p* Value	FC qRT-PCR **	*p* Value	FC Microarray *	Adjusted *p* Value	FC qRT-PCR	*p* Value
*IL-1β*	11.37	6.27 × 10^−13^	6.08	9 × 10^−4^	9.56	6.01 × 10^−12^	4.96	0.0089
*IL-22*	5.62	3.24 × 10^−8^	5.88	0.0791	5.56	7.73 × 10^−8^	3.65	0.0964
*IL8*	2.22	0.0081	3.13	1.8 × 10^−4^	2.95	4.45 × 10^−4^	2.91	0.0025
*IL17A*	2.28	2.4 × 10^−4^	7.62	0.0022	1.81	0.0025	1.75	0.018
*TNF-α*	2.07	1.82 × 10^−7^	2.99	0.0085	1.71	6.14 × 10^−5^	1.61	0.01

* Fold change compared to DMSO control in the microarray experiment. ** Fold change compared to DMSO control in the qRT-PCR experiment.

## Supplementary Materials

The following are available online at http://www.mdpi.com/2072-6651/10/5/199/s1, Table S1: Expression of the genes involved in the NF-κB signalling pathway, Table S2: List of probes with a fold change >2 in the DON and DON+*S.c.* boulardii conditions, Table S3: Primer sequences used for qRT-PCR analysis (F: Forward; R: Reverse), Figure S1: Comparison between microarray-based Log2 (FC) and qRT-PCR-based results Log2 (2-∆∆Ct) by Spearman correlation scattered plots.
