# Supplementary material for: Saccharomyces cerevisiae boulardii Reduces the Deoxynivalenol-Induced Alteration of the Intestinal Transcriptome
Source: Toxins (Basel). 2018 May 15;10(5):199. doi: 10.3390/toxins10050199 (PMC5983255; doi:10.3390/toxins10050199)
Supplement: Supplementary file 1 [file toxins-10-00199-s001.pdf]

Supplementary Materials: *Saccharomyces cerevisiae* Boulardii Reduces the Deoxynivalenol-Induced Alteration of the Intestinal Transcriptome

Imourana Alassane-Kpembi, Philippe Pinton, Jean-François Hupé, Manon Neves, Yannick Lippi, Sylvie Combes, Mathieu Castex and Isabelle P. Oswald

Table S1: Expression of the genes involved in the NF-κB signalling pathway.

Genes in the NF-κB Signalling pathway	Fold change DON compared to control	Fold change DON+S. c. boulardii compared to control
IL1B	11.37	9.56
IL1A	4.17	3.96
TNFAIP3	3.47	2.78
TNF	2.37	2.08
BMP2	2.04	1.70
NFKBIA	2.00	1.53
Bcl10	1.38	1.41
NFKBIE	1.34	1.53
PELI1	1.50	1.23
NF-κB1	1.41	1.30
IL1R1	1.44	-
TRAF6	1.30	-
*NF-κB2/p52_*	1.39	-
TANK	-	1.18
EP300	1.35	-
FADD	1.16	-
CD40	1.31	-
TNFRSF1A	1.27	-
TNFSF13B	1.24	-
TRAF3	1.23	-
PTPN11	1.23	-
JNK1	-	-
PIK3C3	1.21	-
TNFRSF1B	1.19	-
GHR	-	1.18
PIK3R5	1.17	-
IRAK4	1.17	-
IKBKB	1.14	-
NFKBIB	1.14	-
p65/RelA	1.13	-
TRADD	0.91	-
IGF2R	0.89	-
CHUK	0.84	-
TIRAP	-	0.84
PIK3CG	-	0.82
FGFR2	-	-
Zap70	0.78	-
MRAS	-	-
ATM	0.73	-
EGFR	-	0.82
INSR	-	0.83
PIK3R3	-	0.79
Caspase_8	-	0.77
RRAS	-	0.80

PDGFRB	-	0.78
BMPR1B	-	-
AKT3	0.69	0.66

Table S2: List of probes with a fold change >2 in the DON and DON+*S.c.* boulardii conditions.

Probe Name	DON Fold change	Adjusted *p* Value	DON+*S. c.* boulardii Fold change	Adjusted *p* Value
47522925	11.44	6.2736×10^{-13}	9.05	8.7897×10^{-12}
IL1B	11.37	6.2736×10^{-13}	9.56	6.0056×10^{-12}
IL1B	7.10	6.2736×10^{-13}	6.07	8.7897×10^{-12}
IL22	5.62	3.236×10^{-08}	5.56	7.7315×10^{-08}
60678608	5.13	4.7401×10^{-08}	5.11	1.1015×10^{-07}
NOR-1	5.09	1.0735×10^{-09}	3.88	7.7315×10^{-08}
47523707	5.02	1.2718×10^{-07}	3.90	4.9499×10^{-06}
PTGS2	5.01	3.6347×10^{-08}	3.96	1.3234×10^{-06}
PTGS2	4.95	3.3706×10^{-08}	3.86	1.496×10^{-06}
PTGS2	4.95	3.8989×10^{-08}	3.90	1.4846×10^{-06}
NOR-1	4.91	3.0555×10^{-10}	3.79	2.8721×10^{-08}
47523565	4.76	1.3392×10^{-09}	3.85	5.4539×10^{-08}
CXCL2	4.65	0.00045897	3.22	0.01419023
CXCL2	4.64	0.00081411	3.17	0.02169822
49274638	4.50	0.00053669	3.24	0.01261354
Il1a	4.17	5.7781×10^{-09}	3.96	2.9018×10^{-08}
CCL20	4.17	8.9573×10^{-08}	3.96	3.8487×10^{-07}
NOR-1	3.99	6.5959×10^{-10}	3.45	1.7563×10^{-08}
CCL20	3.95	1.3632×10^{-07}	3.78	4.9146×10^{-07}
66793464	3.94	1.4181×10^{-07}	3.78	5.0347×10^{-07}
IL1A	3.84	7.4723×10^{-09}	3.34	1.1015×10^{-07}
47522877	3.78	9.7837×10^{-09}	3.51	6.5873×10^{-08}
93205063	3.75	8.4381×10^{-09}	2.93	7.0177×10^{-07}
CN156953	3.62	1.8359×10^{-09}	3.10	5.4539×10^{-08}
TC627308	3.57	2.6667×10^{-07}	3.10	4.3921×10^{-06}
gi	3.47	1.0808×10^{-08}	2.78	8.1471×10^{-07}
47523041	3.46	4.1671×10^{-06}	2.82	0.00011743
IL1A	3.39	4.5739×10^{-09}	3.17	2.9018×10^{-08}
AW231940	3.39	1.4181×10^{-07}	2.65	1.3585×10^{-05}
AK351795	3.36	5.7781×10^{-09}	2.91	1.2324×10^{-07}
CCR7	3.36	1.3632×10^{-07}	2.63	1.3511×10^{-05}
HAMP	3.34	2.4568×10^{-05}	2.48	0.00195421
gi	3.33	2.6576×10^{-09}	2.65	3.0711×10^{-07}
PLK2	3.28	1.0225×10^{-11}	2.60	2.3193×10^{-09}
AREG	3.26	7.4898×10^{-08}	2.75	2.497×10^{-06}
AREG	3.25	5.0233×10^{-08}	2.72	2.0782×10^{-06}
115551839	3.20	5.2526×10^{-06}	2.95	3.2479×10^{-05}
AREG	3.18	7.0086×10^{-08}	2.87	7.483×10^{-07}
47523807	3.17	2.4638×10^{-08}	2.79	4.8067×10^{-07}
PRDM1	3.17	6.2736×10^{-13}	2.86	8.7897×10^{-12}
PLK2	3.15	1.2924×10^{-11}	2.50	3.8877×10^{-09}
FOSL1	3.11	4.7056×10^{-05}	2.82	0.00026329
TC525635	3.10	4.6374×10^{-10}	2.60	2.9018×10^{-08}
AK348278	3.05	1.0225×10^{-11}	2.60	7.9456×10^{-10}
IL-1beta	3.03	5.9125×10^{-10}	2.59	2.9018×10^{-08}

AJ957055	2.94	1.2365×10^{-08}	2.94	2.9018×10^{-08}
CSF2	2.93	4.5981×10^{-05}	2.30	0.00221347
IL-1beta	2.93	1.6271×10^{-08}	2.81	7.7315×10^{-08}
Adamts1	2.92	9.3286×10^{-10}	2.28	3.2459×10^{-07}
AK347702	2.91	0.00011255	2.34	0.0033115
CRSP-2	2.90	1.3083×10^{-05}	2.04	0.00456955
ADAMTS1	2.90	2.0854×10^{-10}	2.11	3.839×10^{-07}
A_72_P320813	2.89	5.7781×10^{-09}	2.64	6.3784×10^{-08}
TC555952	2.87	0.00010277	3.32	2.7122×10^{-05}
OTTSUST00000000991	2.87	9.2221×10^{-05}	2.23	0.00441692
ADAMTS1	2.85	4.8823×10^{-07}	1.76	0.00609687
PRDM1	2.84	6.9286×10^{-11}	2.59	9.8395×10^{-10}
ENSSSCT00000001477	2.75	6.6238×10^{-05}	2.38	0.00086986
CD274	2.74	3.4727×10^{-07}	1.89	0.00074745
RRAD	2.73	0.00150172	2.08	0.03544042
PRDM1	2.73	9.3632×10^{-11}	2.55	9.8395×10^{-10}
LOC100125547	2.71	3.6493×10^{-06}	1.81	0.00714535
68534987	2.71	1.1113×10^{-06}	1.98	0.0006497
gi	2.69	1.1126×10^{-07}	2.53	7.3347×10^{-07}
55926204	2.68	9.7709×10^{-09}	2.33	3.2936×10^{-07}
IFRD1	2.66	1.4724×10^{-08}	2.32	4.8577×10^{-07}
HAMP	2.64	0.00012804	2.16	0.00378074
GADD45G	2.64	7.5736×10^{-06}	2.70	9.5435×10^{-06}
IL8	2.63	0.00014637	2.85	7.8232×10^{-05}
atf3	2.61	1.5184×10^{-06}	2.57	4.0408×10^{-06}
47523123	2.60	0.00019864	2.81	0.0001057
115547203	2.58	6.9675×10^{-08}	2.23	3.0495×10^{-06}
Gadd45b	2.58	0.00022465	2.11	0.00621803
SELE	2.58	4.8509×10^{-05}	1.94	0.00663333
gi	2.57	2.5555×10^{-07}	2.59	4.7838×10^{-07}
TC549648	2.55	5.1169×10^{-06}	2.63	5.512×10^{-06}
47523601	2.54	0.00014383	1.94	0.01214024
113205835	2.54	1.5531×10^{-06}	2.67	1.3124×10^{-06}
GADD45A	2.52	1.7789×10^{-06}	2.58	2.497×10^{-06}
TC597844	2.52	1.272×10^{-07}	2.25	2.7958×10^{-06}
SOCS3	2.51	1.6249×10^{-07}	1.96	6.138×10^{-05}
IL8	2.51	0.00026723	2.81	8.8101×10^{-05}
SELE	2.51	0.00021952	1.99	0.00988219
47523021	2.50	1.5379×10^{-07}	2.02	2.95×10^{-05}
IL8	2.49	0.00034265	2.91	6.6146×10^{-05}
CXCL2	2.49	0.01969183	1.94	0.10950617
50979294	2.48	2.5245×10^{-07}	1.87	0.00020473
Csf2	2.48	0.00022597	1.95	0.0118176
ADM	2.46	2.0241×10^{-07}	2.02	2.7197×10^{-05}
BTG2	2.45	9.0718×10^{-07}	2.32	5.6846×10^{-06}
GADD45A	2.44	3.3295×10^{-06}	2.60	2.1169×10^{-06}
TC625160	2.43	3.1994×10^{-07}	2.04	2.8814×10^{-05}
MMP12	2.41	0.00020527	2.07	0.00352334
SELE	2.40	0.00017649	1.92	0.00894318

LOC100153365	2.40	0.0001111	2.34	0.00026507
47523043	2.39	9.5678×10^{-06}	1.83	0.00254017
47523385	2.39	2.1361×10^{-08}	2.15	4.8067×10^{-07}
PMAIP1	2.39	1.9223×10^{-08}	2.19	3.097×10^{-07}
RND1	2.39	3.3366×10^{-06}	2.48	3.073×10^{-06}
PRDM1	2.38	6.9286×10^{-11}	2.18	1.3328×10^{-09}
gi	2.38	2.7814×10^{-08}	2.39	5.9161×10^{-08}
OTTSUST00000000652	2.37	7.754×10^{-07}	2.08	2.5923×10^{-05}
socs3	2.37	5.9669×10^{-07}	1.89	0.00015742
LOC100522679	2.37	9.2332×10^{-09}	1.99	1.1332×10^{-06}
ADM	2.36	9.5664×10^{-07}	2.15	1.3585×10^{-05}
gi	2.33	3.4727×10^{-07}	2.24	1.7316×10^{-06}
AK347353	2.31	0.00506051	1.85	0.05661784
RGS1	2.30	5.9024×10^{-07}	2.27	1.6038×10^{-06}
ARG1	2.29	0.00018498	2.33	0.00023838
IL17A	2.28	0.00257817	1.95	0.02535734
gi	2.26	5.1964×10^{-07}	2.02	1.2557×10^{-05}
ENSSSCT00000005051	2.24	8.092×10^{-07}	1.80	0.00023044
gi	2.24	0.03555443	1.91	0.11393206
AK347353	2.23	0.0029344	1.80	0.0481684
IFRD1	2.23	1.3034×10^{-06}	2.08	1.2461×10^{-05}
gi	2.23	4.8086×10^{-07}	1.71	0.00048171
PMAIP1	2.22	4.8815×10^{-08}	2.00	1.3997×10^{-06}
RGS1	2.22	1.9239×10^{-06}	2.26	2.874×10^{-06}
IL8	2.21	0.00814111	2.95	0.0004445
BTG2	2.21	1.4181×10^{-07}	1.80	4.8351×10^{-05}
TC527871	2.21	9.8502×10^{-05}	1.67	0.01968218
CXCR4	2.20	8.1533×10^{-07}	2.00	1.4729×10^{-05}
stc1	2.20	8.092×10^{-07}	1.69	0.00072931
CXCR4	2.19	7.3317×10^{-07}	1.88	5.1053×10^{-05}
NEDD9	2.19	6.689×10^{-08}	2.12	3.1774×10^{-07}
BIRC3	2.18	1.7789×10^{-06}	1.80	0.0002492
STC1	2.18	2.4211×10^{-06}	1.71	0.00095903
TC614762	2.17	9.7837×10^{-09}	1.68	2.3958×10^{-05}
BTG2	2.16	2.2905×10^{-07}	1.76	7.627×10^{-05}
TNF	2.15	7.8791×10^{-07}	1.82	7.6972×10^{-05}
NEDD9	2.15	8.9573×10^{-08}	2.14	2.3285×10^{-07}
GADD45B	2.15	0.00234554	1.58	0.09308427
NR4A2	2.15	5.3429×10^{-07}	1.93	1.3568×10^{-05}
47523273	2.14	4.8388×10^{-06}	2.10	1.3585×10^{-05}
TC564655	2.14	4.8952×10^{-06}	1.73	0.00101017
STC1	2.14	9.3229×10^{-07}	1.73	0.00029361
TSC22D2	2.13	1.3119×10^{-06}	1.76	0.00021609
115548802	2.13	0.00293895	1.63	0.07359836
SPRY2	2.10	2.2798×10^{-08}	1.99	2.0813×10^{-07}
NP276701	2.09	0.00074132	1.60	0.05170575
TC617997	2.08	8.1358×10^{-06}	2.07	1.7259×10^{-05}
49618793	2.08	3.1885×10^{-09}	1.62	9.9325×10^{-06}
PDE4B	2.07	1.1412×10^{-06}	1.48	0.01036891

GEM	2.07	5.1169×10^{-06}	1.77	0.00032865
TNF	2.07	1.8216×10^{-07}	1.71	6.138×10^{-05}
LOC396677	2.06	4.3308×10^{-05}	2.07	6.5829×10^{-05}
LOC396677	2.06	7.0174×10^{-05}	2.13	6.2581×10^{-05}
CD83	2.06	3.4839×10^{-07}	1.65	0.00022829
CALCB	2.06	0.00013461	1.59	0.02424988
A_72_P164316	2.04	1.1394×10^{-08}	1.70	4.556×10^{-06}
BDKRB1	2.04	0.01829254	1.60	0.14293007
LOC100152253	2.04	3.6397×10^{-05}	1.86	0.00039613
PDE4B	2.03	1.5677×10^{-06}	1.78	8.2115×10^{-05}
dusp6	2.01	5.9436×10^{-06}	1.78	0.00019727
TNF	2.01	8.0164×10^{-06}	1.74	0.00043955
FST	2.01	6.1328×10^{-05}	1.88	0.00040132
BMP2	2.01	9.7709×10^{-09}	1.65	6.758×10^{-06}
TC572412	2.01	0.00445372	1.79	0.03049821
52346211	2.00	1.5483×10^{-06}	1.53	0.00324983
IL17A	2.00	0.00023512	1.82	0.00248978
ifrd1	2.00	3.8407×10^{-06}	1.65	0.00085465

Table S3: Primer sequences used for qRT-PCR analysis (F: Forward; R: Reverse).

Gene symbol	Gene name	Primer sequence	Temperature (°C)	Product size (bp)	References
CycloA	Cyclophilin A	F: CCCACCGTCTTCTTCGACAT	83	92	NM_214353
		R: TCTGCTGTCTTTGGAACTTTGTCT			[1]
RPL32	Ribosomal protein L32	F: AGTTCATCCGGCACCAGTCA	80	92	NM_001001636
		R: GAACCTTCTCCGCACCCTGT			[1]
IL1-α	Interleukin 1- alpha	F: TCAGCCGCCCATCCA	79	68	NM_214029.1
		R: AGCCCCCGGTGCCATGT			[2]
IL1-β	Interleukin 1 - beta	F: ATGCTGAAGGCTCTCCACCTC	78	89	NM_214055
		R: TTGTTGCTATCATCTCCTTGCAC			[1]
IL12p40	Interleukin 12 - p 40	F: GGTTTCAGACCCGACGAACTCT	82.5	112	NM_214013
		R : CATATGGCCACAATGGGAGATG			[2]
IL17A	Interleukin 17 - alpha	F: CCAGACGGCCCTCAGATTAC	79.6	66	AB102693
		R: CACTTGGCCTCCCAGATCAC			[2]
IL22	Interleukin - 22	F: AAGCAGGTCCTGAACTTCAC	81.3	133	AY937228
		R: CACCCTTAATACGGCATTGG			[2]
TNF-α	Tumor necrosis factor - alpha	F: ACTGCACTTCGAGGTTATCGG	82	118	NM_214022
		R: GGCGACGGGCTTATCTGA			[2]
IL-8	Interleukin - 8	F: GCTCTCTGTGAGGCTGCAGTTC	79	79	NM_213867
		R: AAGGTGTGGAATGCGTATTTATGC			[3]
CAT	Catalase	F: TGACAAGATGCTGCAGGGC	80	55	NM_214301
		R: GCGGTGGCGGTGAGTG			Current study
LXR	Liver X receptor	F: CCTGGGCGCTCGGAC	79	68	XM_005660919.1
		R: CCTCCACCCACAAGGACATCT			Current study
Cldn4	Claudin - 4	F: CTGCTTTGCTGCAACTGCC	85	107	AK233156
		R: TCAACGGTAGCACCTTACACGTAGT			Current study
LYZ	Lysozyme	F: GGTCTATGATCGGTGCGAGTTC	77	51	ENSSSCT00000034939
		R: TCCATGCCAGACTTTTTCAGAAT			[1]
NF-κB	Nuclear factor kappa B	F: CCTCCACAAGGCAGCAAATAG	79	61	ENSSSCT00000033438
		R: TCCACACCGCTGTCACAGA			[4]

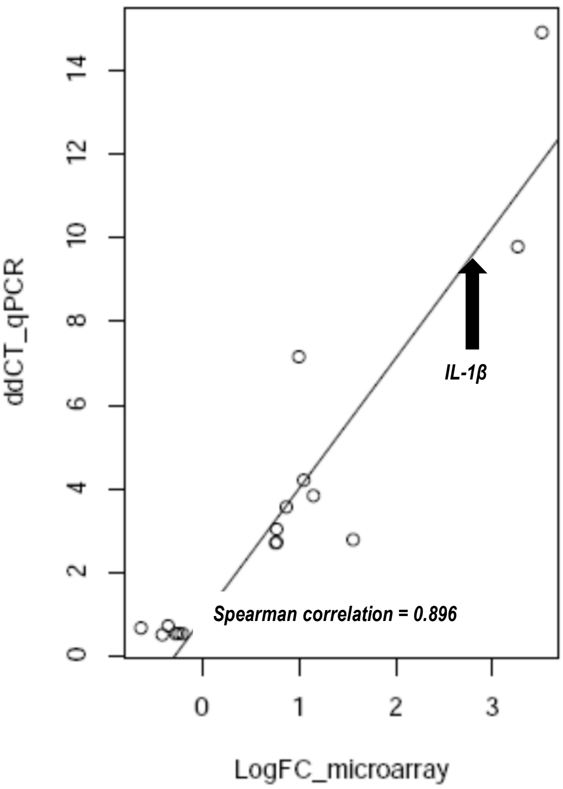

Figure S1: Comparison between microarray-based results (Log2(FC)) and qRT-PCR-based results Log2 (2-ΔΔCt) by Spearman correlation scattered plots.

References

1. Gourbeyre, P.; Berri, M.; Lippi, Y.; Meurens, F.; Vincent-Naulleau, S.; Laffitte, J.; Rogel-Gaillard, C.; Pinton, P.; Oswald, I.P. Pattern recognition receptors in the gut: Analysis of their expression along the intestinal tract and the crypt/villus axis. *Physiol Rep* **2015**, *3*, e12225.
2. Cano, P.M.; Seeboth, J.; Meurens, F.; Cognie, J.; Abrami, R.; Oswald, I.P.; Guzylack-Piriou, L. Deoxynivalenol as a new factor in the persistence of intestinal inflammatory diseases: An emerging hypothesis through possible modulation of th17-mediated response. *PloS one* **2013**, *8*, e53647.
3. Alassane-Kpembi, I.; Puel, O.; Pinton, P.; Cossalter, A.M.; Chou, T.C.; Oswald, I.P. Co-exposure to low doses of the food contaminants deoxynivalenol and nivalenol has a synergistic inflammatory effect on intestinal explants. *Arch Toxicol* **2017**, *91*, 2677-2687.
4. Alassane-Kpembi, I.; Gerez, J.R.; Cossalter, A.M.; Neves, M.; Laffitte, J.; Naylies, C.; Lippi, Y.; Kolf-Clauw, M.; Bracarense, A.P.L.; Pinton, P., *et al.* Intestinal toxicity of the type b trichothecene mycotoxin fusarenon-x: Whole transcriptome profiling reveals new signaling pathways. *Sci Rep* **2017**, *7*, 7530.
